# 2-(4-Nitro­phen­yl)-4,5-diphenyl-1*H*-imidazol-3-ium chloride

**DOI:** 10.1107/S1600536809020807

**Published:** 2009-06-06

**Authors:** Yi Zhang

**Affiliations:** a Ordered Matter Science Research Center, College of Chemistry and Chemical Engineering, Southeast University, Nanjing 210096, People’s Republic of China, and Department of Physics, Southeast University, Nanjing 210096, People’s Republic of China

## Abstract

In the cation of the title compound, C_21_H_16_N_3_O_2_^+^·Cl^−^, the N atom in the 3-position of the imidazole ring is protonated. The three pendant aromatic rings are twisted from the plane of the imidazolium ring by dihedral angles of 31.69 (14)°, 31.09 (14)° and 34.77 (14)°. In the crystal structure, N—H⋯Cl hydrogen bonds link the mol­ecules, forming a chain parallel to the *b* axis.

## Related literature

For uses of imidazole derivatives, see: Dai & Fu (2008 ▶); Fu *et al.* (2008 ▶); Huang *et al.* (2008 ▶). 
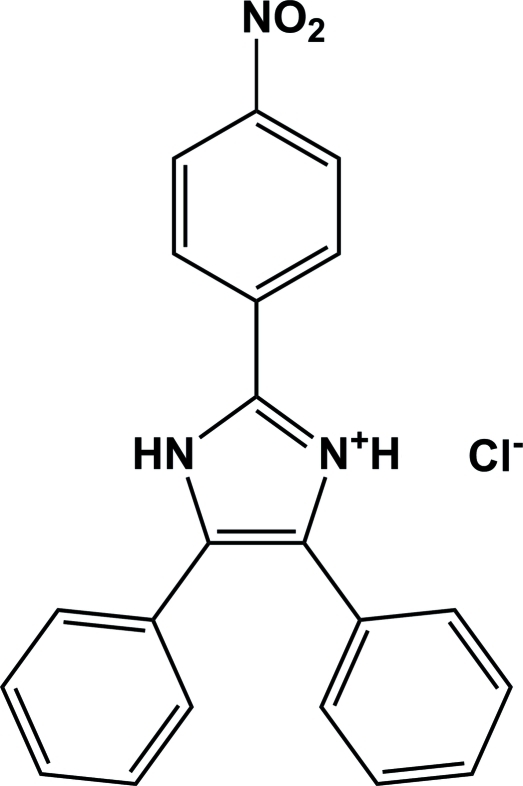


## Experimental

### 

#### Crystal data

C_21_H_16_N_3_O_2_^+^·Cl^−^
*M* *_r_* = 377.82
Monoclinic, 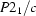

*a* = 15.106 (3) Å
*b* = 15.837 (3) Å
*c* = 7.8833 (16) Å
β = 105.10 (3)°
*V* = 1820.8 (7) Å^3^
*Z* = 4
Mo *K*α radiation
μ = 0.23 mm^−1^
*T* = 298 K
0.45 × 0.40 × 0.25 mm

#### Data collection

Rigaku Mercury2 diffractometer
Absorption correction: multi-scan (*CrystalClear*; Rigaku, 2005 ▶) *T*_min_ = 0.910, *T*_max_ = 1.000 (expected range = 0.859–0.944)
18591 measured reflections
4165 independent reflections
2393 reflections with *I* > 2σ(*I*)
*R*_int_ = 0.109

#### Refinement

*R*[*F* ^2^ > 2σ(*F* ^2^)] = 0.062
*wR*(*F* ^2^) = 0.157
*S* = 1.03
4165 reflections
245 parameters
H-atom parameters constrained
Δρ_max_ = 0.32 e Å^−3^
Δρ_min_ = −0.35 e Å^−3^

### 

Data collection: *CrystalClear* (Rigaku, 2005 ▶); cell refinement: *CrystalClear*; data reduction: *CrystalClear*; program(s) used to solve structure: *SHELXS97* (Sheldrick, 2008 ▶); program(s) used to refine structure: *SHELXL97* (Sheldrick, 2008 ▶); molecular graphics: *SHELXTL* (Sheldrick, 2008 ▶); software used to prepare material for publication: *SHELXTL*.

## Supplementary Material

Crystal structure: contains datablocks I, New_Global_Publ_Block. DOI: 10.1107/S1600536809020807/ci2820sup1.cif

Structure factors: contains datablocks I. DOI: 10.1107/S1600536809020807/ci2820Isup2.hkl

Additional supplementary materials:  crystallographic information; 3D view; checkCIF report

## Figures and Tables

**Table 1:** Hydrogen-bond geometry (Å, °)

*D*—H⋯*A*	*D*—H	H⋯*A*	*D*⋯*A*	*D*—H⋯*A*
N1—H1*A*⋯Cl1	0.86	2.25	3.105 (2)	176
N2—H2*A*⋯Cl1^i^	0.86	2.28	3.139 (2)	175

Symmetry code: (i) 
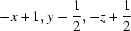
.

**Fig. 1.**
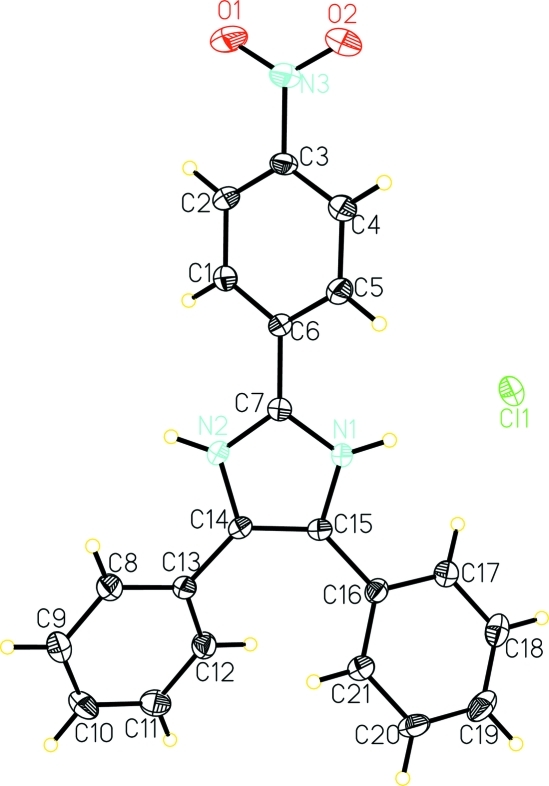
A view of the title compound with the atomic numbering scheme. Displacement ellipsoids are drawn at the 30% probability level.

**Fig. 2.**
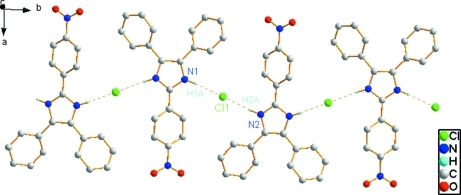
The crystal packing of the title compound viewed along the c axis, showing a hydrogen bonded chain. H atoms not involved in hydrogen bonding (dashed lines) have been omitted for clarity.

## supplementary crystallographic information

### Comment

Imidazole derivatives have found wide range of applications in coordination chemistry because of their multiple coordination modes as ligands to metal ions and for the construction of novel metal-organic frameworks (Huang *et al.* 2008; Fu *et al.* 2008; Dai & Fu 2008). We report herein the crystal structure of the title compound, 2-(4-nitrophenyl)-4,5-diphenyl-1*H*-imidazol-3-ium chloride.

The title compound contains an organic cation and a Cl^-^ anion in the asymmetric unit. The imidazole N atom in 3-position is protonated. The C1-C6, C8-C13 and C16-C21 benzene rings are twisted from the imidazolium ring an angle of 31.69 (14)°, 31.09 (14)° and 34.77 (14)°, respectively.

The crystal packing is stabilized by N—H···Cl hydrogen bonds to form a linear chain parallel to the *b* axis (Table 1 and Fig. 2).

### Experimental

Under nitrogen protection, 1,2-diphenyl-ethane-1,2-dione (20 mmol), 4-nitrobenzaldehyde (20 mmol) and amine acetate (50 mmol) were dissolved in 60 ml of HOAc. The mixture was stirred at 383 K for 20 h. The resulting solution was poured into ice water (200 ml) and after neutralizing the mixture with NaOH (6 mol/L) a white solid was obtained. After filtration and washing with distilled water the crude product was recrystallized from a ethanolic solution (150 ml) to which HCl (5 ml) was added to yield colourless block-shaped crystals of the title compound, suitable for X-ray analysis.

### Refinement

H atoms attached to C and N atoms were positioned geometrically and treated as riding, with C-H = 0.93 Å, N-H = 0.86 Å and *U*_iso_(H) = 1.2U_eq_(C,N).

### Crystal data

**Table:**

C_21_H_16_N_3_O_2_^+^·Cl^−^	*F*(000) = 784
*M**_r_* = 377.82	*D*_x_ = 1.378 Mg m^−^^3^
Monoclinic, *P*2_1_/*c*	Mo *K*α radiation, λ = 0.71073 Å
Hall symbol: -P 2ybc	Cell parameters from 4160 reflections
*a* = 15.106 (3) Å	θ = 3.1–27.5°
*b* = 15.837 (3) Å	µ = 0.23 mm^−^^1^
*c* = 7.8833 (16) Å	*T* = 298 K
β = 105.10 (3)°	Block, colourless
*V* = 1820.8 (7) Å^3^	0.45 × 0.40 × 0.25 mm
*Z* = 4	

### Data collection

**Table:**

Rigaku Mercury2 diffractometer	4165 independent reflections
Radiation source: fine-focus sealed tube	2393 reflections with *I* > 2σ(*I*)
graphite	*R*_int_ = 0.109
Detector resolution: 13.6612 pixels mm^-1^	θ_max_ = 27.5°, θ_min_ = 3.1°
CCD profile fitting scans	*h* = −19→19
Absorption correction: multi-scan (*CrystalClear*; Rigaku, 2005)	*k* = −20→20
*T*_min_ = 0.910, *T*_max_ = 1.000	*l* = −10→10
18591 measured reflections	

### Refinement

**Table:**

Refinement on *F*^2^	Secondary atom site location: difference Fourier map
Least-squares matrix: full	Hydrogen site location: inferred from neighbouring sites
*R*[*F*^2^ > 2σ(*F*^2^)] = 0.062	H-atom parameters constrained
*wR*(*F*^2^) = 0.157	*w* = 1/[σ^2^(*F*_o_^2^) + (0.0533*P*)^2^ + 0.4461*P*] where *P* = (*F*_o_^2^ + 2*F*_c_^2^)/3
*S* = 1.03	(Δ/σ)_max_ = 0.001
4165 reflections	Δρ_max_ = 0.32 e Å^−^^3^
245 parameters	Δρ_min_ = −0.35 e Å^−^^3^
0 restraints	Extinction correction: *SHELXL97*, Fc^*^=kFc[1+0.001xFc^2^λ^3^/sin(2θ)]^-1/4^
Primary atom site location: structure-invariant direct methods	Extinction coefficient: 0.0053 (10)

### Special details

**Table:**

Geometry. All e.s.d.'s (except the e.s.d. in the dihedral angle between two l.s. planes) are estimated using the full covariance matrix. The cell e.s.d.'s are taken into account individually in the estimation of e.s.d.'s in distances, angles and torsion angles; correlations between e.s.d.'s in cell parameters are only used when they are defined by crystal symmetry. An approximate (isotropic) treatment of cell e.s.d.'s is used for estimating e.s.d.'s involving l.s. planes.
Refinement. Refinement of *F*^2^ against ALL reflections. The weighted *R*-factor *wR* and goodness of fit *S* are based on *F*^2^, conventional *R*-factors *R* are based on *F*, with *F* set to zero for negative *F*^2^. The threshold expression of *F*^2^ > σ(*F*^2^) is used only for calculating *R*-factors(gt) *etc*. and is not relevant to the choice of reflections for refinement. *R*-factors based on *F*^2^ are statistically about twice as large as those based on *F*, and *R*- factors based on ALL data will be even larger.

### Fractional atomic coordinates and isotropic or equivalent isotropic displacement parameters (Å^2^)

**Table:**

	*x*	*y*	*z*	*U*_iso_*/*U*_eq_	
Cl1	0.50431 (5)	0.90216 (4)	0.28931 (13)	0.0625 (3)	
N1	0.57993 (14)	0.72042 (13)	0.2790 (3)	0.0325 (5)	
H1A	0.5620	0.7717	0.2840	0.039*	
C7	0.52567 (17)	0.65254 (16)	0.2629 (3)	0.0321 (6)	
N2	0.57744 (14)	0.58548 (13)	0.2592 (3)	0.0339 (5)	
H2A	0.5581	0.5342	0.2501	0.041*	
N3	0.14392 (17)	0.65834 (17)	0.2081 (4)	0.0480 (7)	
C6	0.42716 (17)	0.65341 (15)	0.2489 (3)	0.0323 (6)	
C2	0.27691 (18)	0.59458 (17)	0.1358 (4)	0.0399 (7)	
H2	0.2382	0.5540	0.0700	0.048*	
C15	0.66881 (17)	0.69658 (16)	0.2865 (3)	0.0307 (6)	
C13	0.73980 (17)	0.54725 (16)	0.2772 (3)	0.0320 (6)	
C3	0.24275 (18)	0.65705 (17)	0.2217 (4)	0.0370 (7)	
C1	0.37003 (18)	0.59320 (16)	0.1493 (4)	0.0389 (7)	
H1	0.3944	0.5517	0.0912	0.047*	
C14	0.66699 (17)	0.60935 (16)	0.2719 (3)	0.0308 (6)	
C18	0.7947 (2)	0.89632 (18)	0.2438 (4)	0.0449 (8)	
H18	0.7821	0.9490	0.1908	0.054*	
C4	0.29746 (19)	0.71829 (17)	0.3209 (4)	0.0418 (7)	
H4	0.2725	0.7601	0.3773	0.050*	
O2	0.11287 (15)	0.71883 (15)	0.2695 (4)	0.0696 (7)	
C5	0.39062 (19)	0.71577 (17)	0.3342 (4)	0.0404 (7)	
H5	0.4291	0.7562	0.4008	0.049*	
O1	0.09771 (15)	0.59864 (15)	0.1374 (4)	0.0740 (8)	
C16	0.74249 (18)	0.75918 (15)	0.3069 (3)	0.0315 (6)	
C17	0.7250 (2)	0.83847 (16)	0.2293 (4)	0.0374 (7)	
H17	0.6657	0.8526	0.1671	0.045*	
C9	0.8082 (2)	0.41135 (18)	0.3652 (4)	0.0455 (7)	
H9	0.8052	0.3581	0.4132	0.055*	
C21	0.83144 (18)	0.74111 (17)	0.4041 (4)	0.0387 (7)	
H21	0.8441	0.6893	0.4608	0.046*	
C12	0.81528 (19)	0.56718 (17)	0.2152 (4)	0.0390 (7)	
H12	0.8172	0.6190	0.1606	0.047*	
C8	0.73622 (19)	0.46743 (17)	0.3498 (4)	0.0399 (7)	
H8	0.6853	0.4518	0.3881	0.048*	
C11	0.8874 (2)	0.51169 (19)	0.2330 (4)	0.0497 (8)	
H11	0.9381	0.5266	0.1934	0.060*	
C20	0.9012 (2)	0.7994 (2)	0.4172 (4)	0.0480 (8)	
H20	0.9605	0.7861	0.4812	0.058*	
C19	0.8833 (2)	0.8769 (2)	0.3361 (4)	0.0501 (8)	
H19	0.9303	0.9157	0.3435	0.060*	
C10	0.8841 (2)	0.4339 (2)	0.3099 (4)	0.0517 (8)	
H10	0.9331	0.3967	0.3243	0.062*	

### Atomic displacement parameters (Å^2^)

**Table:**

	*U*^11^	*U*^22^	*U*^33^	*U*^12^	*U*^13^	*U*^23^
Cl1	0.0444 (5)	0.0305 (4)	0.1135 (8)	0.0066 (3)	0.0224 (5)	0.0007 (4)
N1	0.0281 (13)	0.0225 (11)	0.0463 (14)	−0.0003 (9)	0.0087 (11)	−0.0024 (10)
C7	0.0270 (14)	0.0291 (14)	0.0390 (15)	0.0003 (12)	0.0064 (12)	−0.0005 (12)
N2	0.0293 (13)	0.0238 (11)	0.0482 (14)	−0.0043 (9)	0.0095 (10)	−0.0023 (10)
N3	0.0286 (14)	0.0472 (16)	0.0670 (17)	0.0050 (13)	0.0103 (13)	0.0089 (14)
C6	0.0252 (14)	0.0280 (13)	0.0437 (16)	−0.0024 (11)	0.0086 (12)	0.0015 (12)
C2	0.0272 (15)	0.0368 (16)	0.0520 (18)	−0.0064 (12)	0.0038 (13)	−0.0048 (14)
C15	0.0238 (15)	0.0315 (14)	0.0362 (15)	−0.0007 (11)	0.0068 (12)	−0.0010 (12)
C13	0.0274 (15)	0.0293 (14)	0.0388 (15)	0.0003 (11)	0.0076 (12)	−0.0029 (12)
C3	0.0225 (14)	0.0376 (15)	0.0494 (17)	0.0035 (12)	0.0066 (13)	0.0107 (14)
C1	0.0328 (16)	0.0290 (14)	0.0538 (18)	0.0004 (12)	0.0094 (13)	−0.0066 (13)
C14	0.0251 (14)	0.0293 (14)	0.0381 (15)	−0.0044 (11)	0.0084 (12)	−0.0005 (12)
C18	0.059 (2)	0.0314 (15)	0.0494 (18)	−0.0075 (15)	0.0227 (16)	−0.0027 (14)
C4	0.0341 (17)	0.0382 (16)	0.0564 (19)	0.0021 (13)	0.0175 (14)	−0.0052 (14)
O2	0.0399 (14)	0.0615 (15)	0.112 (2)	0.0115 (12)	0.0282 (14)	−0.0054 (14)
C5	0.0333 (17)	0.0343 (15)	0.0530 (18)	−0.0049 (12)	0.0102 (14)	−0.0111 (14)
O1	0.0313 (13)	0.0674 (16)	0.118 (2)	−0.0119 (12)	0.0094 (13)	−0.0165 (16)
C16	0.0311 (15)	0.0285 (13)	0.0371 (15)	−0.0045 (11)	0.0129 (12)	−0.0038 (12)
C17	0.0371 (17)	0.0324 (14)	0.0428 (16)	−0.0011 (12)	0.0109 (13)	−0.0009 (13)
C9	0.0491 (19)	0.0328 (15)	0.0538 (19)	0.0065 (14)	0.0122 (15)	0.0061 (14)
C21	0.0314 (16)	0.0374 (15)	0.0459 (17)	−0.0038 (13)	0.0072 (13)	−0.0037 (13)
C12	0.0386 (17)	0.0328 (15)	0.0484 (18)	−0.0005 (13)	0.0166 (14)	0.0022 (13)
C8	0.0364 (17)	0.0351 (15)	0.0496 (18)	0.0017 (13)	0.0139 (14)	0.0021 (13)
C11	0.0343 (17)	0.0530 (19)	0.067 (2)	0.0031 (15)	0.0235 (16)	−0.0019 (17)
C20	0.0266 (16)	0.054 (2)	0.060 (2)	−0.0079 (14)	0.0054 (14)	−0.0087 (16)
C19	0.047 (2)	0.0476 (18)	0.063 (2)	−0.0217 (15)	0.0264 (17)	−0.0146 (16)
C10	0.0420 (19)	0.0477 (19)	0.064 (2)	0.0179 (15)	0.0107 (16)	0.0036 (16)

### Geometric parameters (Å, °)

**Table:**

N1—C7	1.338 (3)	C18—C19	1.380 (4)
N1—C15	1.381 (3)	C18—H18	0.93
N1—H1A	0.86	C4—C5	1.384 (4)
C7—N2	1.324 (3)	C4—H4	0.93
C7—C6	1.463 (4)	C5—H5	0.93
N2—C14	1.383 (3)	C16—C17	1.392 (3)
N2—H2A	0.86	C16—C21	1.392 (4)
N3—O1	1.220 (3)	C17—H17	0.9300
N3—O2	1.221 (3)	C9—C10	1.375 (4)
N3—C3	1.469 (4)	C9—C8	1.384 (4)
C6—C1	1.385 (4)	C9—H9	0.93
C6—C5	1.388 (4)	C21—C20	1.384 (4)
C2—C3	1.373 (4)	C21—H21	0.93
C2—C1	1.383 (4)	C12—C11	1.378 (4)
C2—H2	0.93	C12—H12	0.93
C15—C14	1.386 (3)	C8—H8	0.93
C15—C16	1.468 (3)	C11—C10	1.379 (4)
C13—C12	1.389 (4)	C11—H11	0.93
C13—C8	1.395 (4)	C20—C19	1.378 (4)
C13—C14	1.468 (3)	C20—H20	0.93
C3—C4	1.378 (4)	C19—H19	0.93
C1—H1	0.93	C10—H10	0.93
C18—C17	1.378 (4)		
			
C7—N1—C15	110.4 (2)	C3—C4—C5	118.1 (3)
C7—N1—H1A	124.8	C3—C4—H4	121.0
C15—N1—H1A	124.8	C5—C4—H4	121.0
N2—C7—N1	107.2 (2)	C4—C5—C6	120.6 (3)
N2—C7—C6	126.9 (2)	C4—C5—H5	119.7
N1—C7—C6	125.8 (2)	C6—C5—H5	119.7
C7—N2—C14	110.6 (2)	C17—C16—C21	118.2 (2)
C7—N2—H2A	124.7	C17—C16—C15	120.6 (2)
C14—N2—H2A	124.7	C21—C16—C15	121.1 (2)
O1—N3—O2	123.8 (3)	C18—C17—C16	120.6 (3)
O1—N3—C3	118.2 (3)	C18—C17—H17	119.7
O2—N3—C3	118.0 (3)	C16—C17—H17	119.7
C1—C6—C5	119.8 (2)	C10—C9—C8	120.4 (3)
C1—C6—C7	120.5 (2)	C10—C9—H9	119.8
C5—C6—C7	119.7 (2)	C8—C9—H9	119.8
C3—C2—C1	118.6 (2)	C20—C21—C16	120.7 (3)
C3—C2—H2	120.7	C20—C21—H21	119.6
C1—C2—H2	120.7	C16—C21—H21	119.6
N1—C15—C14	105.7 (2)	C11—C12—C13	121.2 (3)
N1—C15—C16	121.4 (2)	C11—C12—H12	119.4
C14—C15—C16	132.9 (2)	C13—C12—H12	119.4
C12—C13—C8	118.3 (2)	C9—C8—C13	120.3 (3)
C12—C13—C14	121.0 (2)	C9—C8—H8	119.9
C8—C13—C14	120.7 (2)	C13—C8—H8	119.9
C2—C3—C4	122.7 (3)	C12—C11—C10	119.8 (3)
C2—C3—N3	118.7 (3)	C12—C11—H11	120.1
C4—C3—N3	118.7 (3)	C10—C11—H11	120.1
C2—C1—C6	120.2 (3)	C19—C20—C21	120.4 (3)
C2—C1—H1	119.9	C19—C20—H20	119.8
C6—C1—H1	119.9	C21—C20—H20	119.8
N2—C14—C15	106.0 (2)	C20—C19—C18	119.3 (3)
N2—C14—C13	122.0 (2)	C20—C19—H19	120.3
C15—C14—C13	131.8 (2)	C18—C19—H19	120.3
C17—C18—C19	120.6 (3)	C9—C10—C11	120.0 (3)
C17—C18—H18	119.7	C9—C10—H10	120.0
C19—C18—H18	119.7	C11—C10—H10	120.0

### Hydrogen-bond geometry (Å, °)

**Table:**

*D*—H···*A*	*D*—H	H···*A*	*D*···*A*	*D*—H···*A*
N1—H1A···Cl1	0.86	2.25	3.105 (2)	176
N2—H2A···Cl1^i^	0.86	2.28	3.139 (2)	175

Symmetry codes: (i) −*x*+1, *y*−1/2, −*z*+1/2.
